# Preferential path attachment model for quantum key distribution networks

**DOI:** 10.1038/s41598-026-43414-x

**Published:** 2026-03-15

**Authors:** Jiří Weiss, Michal Lucki, Radek Mařík, Leoš Boháč

**Affiliations:** Department of Telecommunication Engineering, Faculty of Electrical Engineering, Czech Technical University in Prague, Prague, Czech Republic

**Keywords:** Engineering, Mathematics and computing, Physics

## Abstract

This paper presents a model for path-based growing network with preferential attachment motivated by the deployment of quantum key distribution networks. The model is based on a network constructed from path segments of $\langle \hbox {n}\rangle$ nodes on average to mimic real-world quantum key distribution network architectures. Using continuum formalism and the rate equation method, we derive degree exponent, exact degree distributions and demonstrate properties similar to random networks. The theoretical framework incorporates preferential attachment with variable crossover rates and strategic shortcuts, the satellite links. The approach is validated through extensive simulations implemented in Python. Key findings reveal that network robustness, measured by critical fraction for giant component loss, increases with crossover rate and number of satellite links but decreases with segment length. Average distance scales logarithmically with network size, directly impacting secret key consumption during relaying processes in quantum key distribution networks. While preferential attachment enhances connectivity, the model network does not achieve ultra-small world properties of scale-free networks that would minimize key consumption, providing insights for designing cost-effective quantum communication infrastructures.

## Introduction

The advancements in quantum technologies have facilitated the large-scale construction of quantum key distribution networks (QKDNs)^1^. QKDNs employ specialized hardware to generate secure key between pairs of devices, commonly referred to as Alice and Bob, by leveraging the principles of quantum mechanics. The protocols utilized for quantum key distribution (QKD) exploit phenomena such as the Heisenberg uncertainty principle^2^ and quantum entanglement^3^.

Commercially available quantum key distribution devices now exhibit performance levels that ensure stable and reliable key generation^4,5^. The QKD protocols used to generate key bits between two users are continually examined for their properties, security, and performance. Security proofs for several protocols^6–9^ and implementations^10,11^ have been established. Commercial QKD devices are typically available in pairs, generating keys between them via a link (an optical fiber or a free space channel) that distributes quantum bits (qubits). The devices extract secure keys from detected qubits through a process of post-processing. The entire process, from qubit transmission to key distillation and final secure key generation, has been thoroughly studied and documented^12^. Therefore, QKD technology can be considered secure and reliable for secure key generation.

A pair of key generating devices cannot be arbitrarily distant from each other due to the limitations imposed by qubit transmission, which suffers from losses caused by fiber attenuation and dispersion. Additionally, qubits cannot be duplicated^13^ and transmitted due to the principles of quantum mechanics. Thus, when the distance between two end nodes exceeds the maximum distance ($\sim 100$ km) of a single QKD link, multiple device pairs are arranged sequentially to extend the connection. Placing two quantum key distribution devices, each belonging to a different QKD link, in one location forms an intermediate node. These intermediate nodes, commonly referred to as trusted nodes, serve as key relay points since each pair of QKD devices generates its own set of secure keys. Trust in these nodes is critical, as their compromise could lead to the disclosure of cryptographic keys used for encryption. Key relaying is essential to transfer the cryptographic key from its origin to its final destination, where the ciphertext is decrypted using the same key in a symmetric cryptosystem, such as AES-256.

Network topology has a direct impact on the amount of key material consumed during the key relaying process in QKD networks. Key relaying involves transport of secure keys used for data encryption and decryption and consumes keys at intermediate trusted nodes. Therefore, it is advantageous to reduce the average distance to minimize the consumption of keys during the key relaying process. However, QKDNs have architectural constraints less explored in network growth models. Network architectures based on trusted intermediate nodes enabling key relaying over extended distances create a path-based deployment structure with distinct growth dynamics and optimization requirements.

QKDNs, like transportation systems, power grids, and communication networks, are carefully planned and optimized for specific objectives rather than following probabilistic attachment rules. Nevertheless, empirical studies reveal that planned infrastructure can exhibit scale-free property. For example, the global airline network shows power-law degree distributions with hub formation^14,15^ despite centralized planning due to economic optimization driving connectivity. This scale-free topology is a characteristic of preferential attachment (PA)^16^, where new arriving nodes prefer high-degree hubs to link to. Given that hub formation reduces average distance and PA mechanism is capable of creating hubs in other networks, we incorporated PA into our QKDN model to explore whether a similar topology can be achieved. To test whether PA produces hub formation in path-based networks, we developed the Preferential Path Attachment (PPA) model. The model grows networks by adding path segments of $\langle n \rangle$ nodes with end nodes connecting to the existing network via PA. The average $\langle n \rangle$ is the mean value of normally distributed path segment lengths $n_i$. In the case of PA prevalence, a hub-dominated scale-free topology that minimizes average distance for efficient key relaying is expected. We derived analytical degree distribution for PPA network and simulated the network in Python to examine its properties such as the average distance between nodes and robustness of the network. Tunable input parameters of the model allow network planners to explore topological consequences of deployment strategies before committing to physical infrastructure.

Despite incorporating PA, the PPA network produces degree exponent $\gamma > 3$, characteristic of random networks rather than scale-free networks due to path constraints fundamentally preventing expected hub formation. Additionally, the simulation incorporates a manually set number of satellite links (shortcuts) in the PPA network by adding an edge in between two randomly chosen nodes to examine their impact on average distance and robustness. The effect of shortcuts on average distance reveal diminishing returns as shortcut density increases.

This paper is arranged as follows: the Key Relaying section elucidates the key relaying method for establishing secure end-to-end communication, and highlights the limitations of the key relaying process, particularly the increased key consumption dependent on the distance between communicating end nodes. The Methods section examines the properties of path and cycle graphs (chain and ring networks) and introduces the formation mechanism and properties of the PPA network. The Results section analyzes the robustness of the presented topologies and the average distance between nodes, including strategic shortcut optimization. The Discussion concludes with broader implications and future directions.

## Key relaying

While key generation is conducted within the quantum layer of the QKDN, key relaying occurs at a higher layer. Once generated in QKD devices, secure keys are managed by the Key Management System (KMS)^17^.

**Fig. 1:**
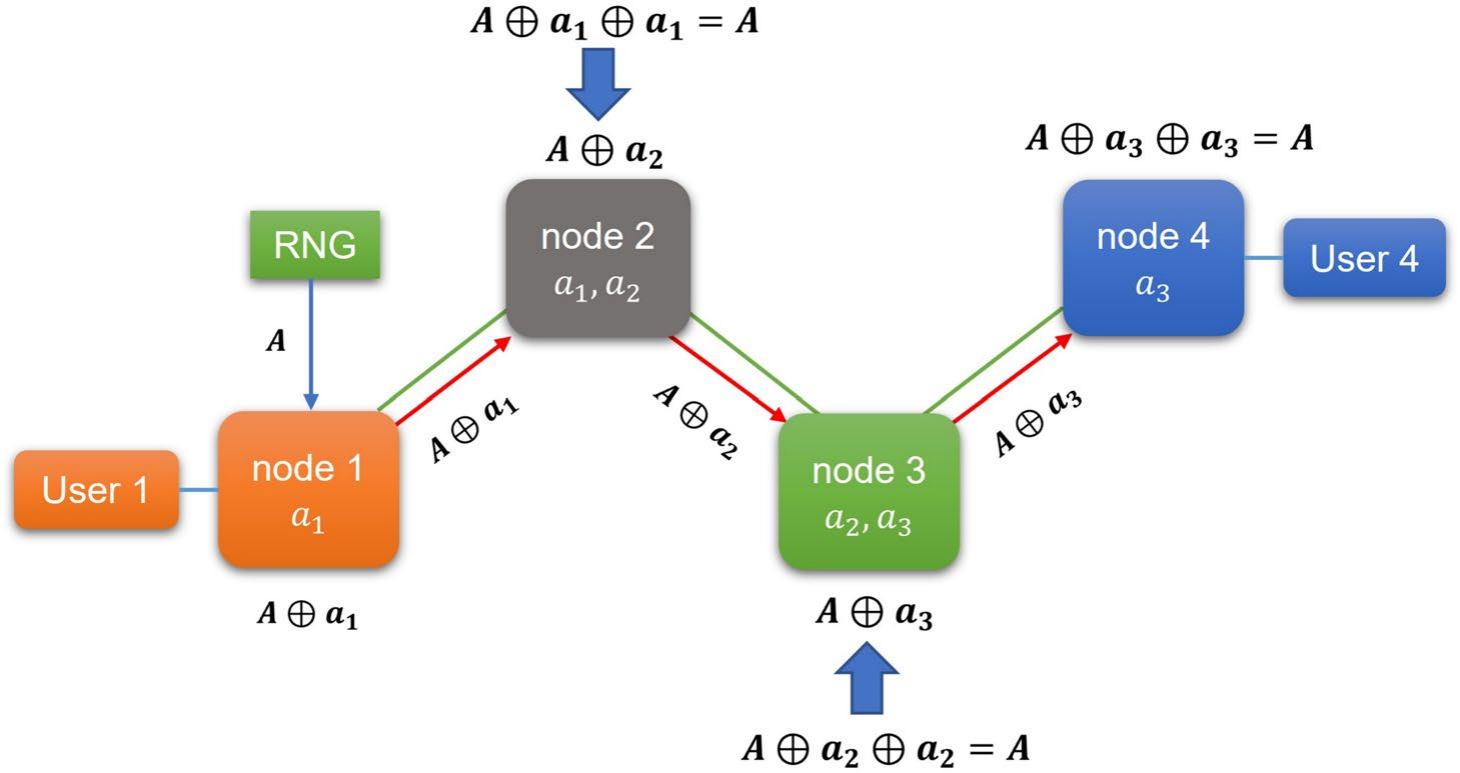
Key hopping using one-time pad encryption in QKDN.

Figure 1 shows the key relaying process where User 1, connected to node 1, intends to transmit an encrypted message to User 4, connected to node 4, using the symmetric cryptosystem AES-256. User 1 and User 4 must share a secret key for plaintext encryption and ciphertext decryption. Given that the distance between the two users surpasses the limit for a single QKD pair to successfully generate a secure key, the network incorporates intermediate relay (trusted) nodes. Key $a_i$ generated by QKD methods can be only produced between neighboring nodes. Key $a_1$ is shared between node 1 and node 2 only, key $a_2$ is shared between node 2 and node 3 only etc. Let the key *A* for plaintext encryption be generated by a random number generator (RNG). To forward the key *A* from node 1 (User 1) to node 4 (User 4) the Vernam cipher (one-time pad (OTP)) is used for key *A* encryption. It represents a solution to the key relaying problem with proven information-theoretic security^18^. The key *A* is encrypted by the exclusive-OR (XOR) operation as $A\oplus a_1$. The result $A\oplus a_1$ is relayed from node 1 to node 2. To move the key *A* to node 3 which possesses keys $a_2$ and $a_3$, key *A* needs to be sent in the form $A\oplus a_2$. The XOR of keys *A* and $a_2$ is obtained using operation performed in node 2 where $A\oplus a_2 = A\oplus a_1 \oplus a_1 \oplus a_2$. Node 3 contains secret key *A* after the second key relaying instance and performs key decryption/encryption using its secure QKD generated key $a_2$ and $a_3$. The result $A\oplus a_3$ is then sent to the final end node 4, where key *A* is exposed and used for ciphertext decryption from User 1. User 4, connected to node 4, is thereby able to obtain the plaintext sent by User 1 from distant node 1. The whole process is known as key hopping^19^. Alternative approaches include hybrid key forwarding^20^, where the source node encrypts a random key *A* using post-quantum cryptographic techniques before standard XOR-based QKD forwarding. This ensures no intermediate node gains access to the forwarded key *A*. From a topology perspective, multi-path key relaying^21^ distributes key material across multiple disjoint paths between communicating nodes, improving the resilience of the key delivery process. Its feasibility depends on the availability of multiple independent paths, directly related to the network formation process analyzed in this work.

### KMS and topology

The KMS, with its key relaying mechanism, can support various QKDNs topologies. However, constraints arise from the perspective of quantum transmission. An optical fiber link between two nodes cannot be arbitrarily long due to increasing losses of quantum bits with distance. Therefore, the insertion of intermediate trusted nodes is necessary to bridge two distant endpoints desiring secure communication. Secure keys are generated between all pairs of nodes in the chain (differing for each pair), and the key for plaintext encryption and ciphertext decryption is relayed from one communicating node to the other. It is reasonable to assume that future QKDNs based on point-to-point connections and trusted nodes will consist of chains of nodes equipped with QKD devices generating secure keys between pairs of devices. This chain topology is currently exploited in long-haul terrestrial QKD networks^22^. Connecting such long-haul QKD network chains will form large-scale QKDNs, which, as the authors expect, would operate as intercity or interstate networks^23^. Therefore, we first investigate influence of single chain topology on networks properties.

Initially, we analyze a simple path, followed by a cycle graph. Subsequently, we present a more complex network topology based on path segments, which are finite chains of nodes. The mechanism of PPA network formation will be elucidated, and its properties will be simulated in Python.

## Methods

### Preferential path attachment model for QKD networks

We consider undirected networks and two simple topologies of QKDNs, path topology and cycle topology, and we will examine the properties of the two approaches in QKDN construction. Let us study a simple path first. Any new node to be added to the network connects to the node with lowest degree *k*. In other words, a newly added node connects to one of the ends of the network. The newly added node connects with one link only, creating an end of the enlarged network. Considering the cycle topology, the newly added node connects with two links to the existing nodes of the cycle and thereby expands the cycle.

The degree distribution $p_k$ of the path network is $p_{k=1}^{\text {path}} = 2/N$, $p_{k=2}^{\text {path}} = (N-2)/N$ and $p_{k>2}^{\text {path}}=0$. The degree distribution $p_k$ of the cycle network is

1 $$\begin{aligned} p_{k}^{\text {cycle}} = {\left\{ \begin{array}{ll} 1 & \text {for } k = 2 \\ 0 & \text {otherwise} \end{array}\right. } \end{aligned}$$

From the network properties perspective, the average distance $\langle d^{\text {path}} \rangle$ for path network is linearly dependent on number of nodes as $\propto \frac{1}{3}N$. For cycle networks, the average distance $\langle d^{\text {cycle}} \rangle$ is proportional to number of network nodes as $\propto \frac{1}{4}N$.

Simple path or cycle topologies do not accurately represent real-world QKDNs, nor are they preferred since both path and cycle graphs are vulnerable to node removal and are easily disconnected^24^. Furthermore, a failure of a link or an intermediate trusted node between two nodes with high data traffic (and consequently high key consumption) in a cycle topology would necessitate key relaying, significantly burdening the remaining network nodes to relay the required volume of secure keys. Therefore, a more complex network is needed to introduce e.g. multi-path key relaying. Additionally, the motivation extends to exploring the possibility of increasing network robustness against node failures or removals.

**Fig. 2:**
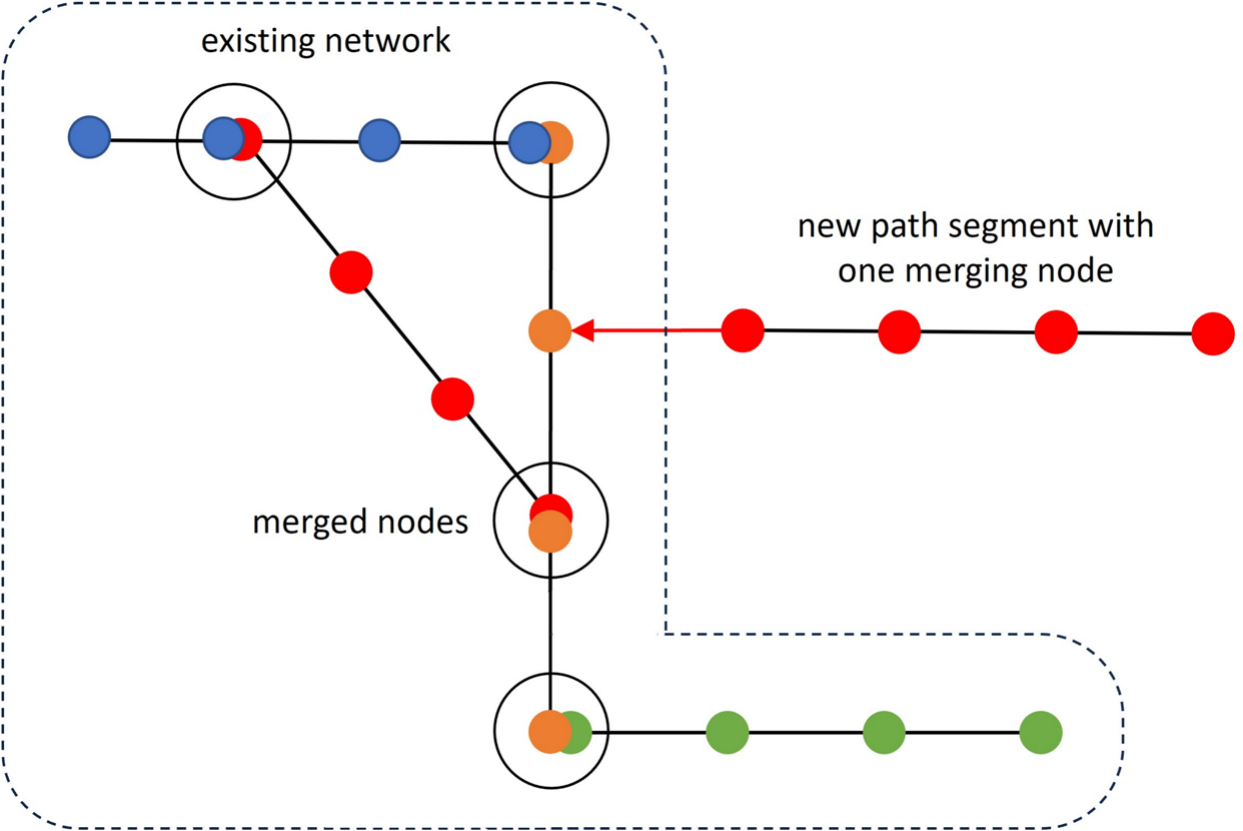
Example of segment based QKDN formation with $\langle n \rangle = 4$ and $\langle x \rangle = 1.25$. Undirected edges for bidirectional key relaying and communication.

A real-world approach to QKDN construction is presented in the following section. It incorporates design of QKDNs with path segments consisting of multiple nodes in series which is a common practice how to connect two distant locations with QKD technology. The PPA model is designed for long-haul path-based QKDNs connecting distant locations, rather than metropolitan mesh-type networks where nodes may be interconnected with less constrained topologies. We will construct the network using simple path segments consisting of $n_i$ nodes each resulting in average of $\langle n \rangle$ nodes over event time *t*. At event time $t=1$, the network will be of one segment only. A new segment will be added to the network at each increment of event time *t*. New path segments will merge the nodes at the segments end(s) with the existing nodes of the network as in Fig. 2.

A new segment of length $n_i$ merges with the existing network through $x_i \in \{1,2\}$ of its end nodes, each forming a trusted node. The average number of merged ends throughout the network is $\langle x \rangle$, which we define as crossover rate. The crossover rate $\langle x \rangle \in [1,2]$ represents the average of $x_i$ across the network. Value $\langle x \rangle =1$ represents a scenario when all segments connect through one end node. Value $\langle x \rangle =1.5$ represents a scenario when new coming segments connect either with one or both end nodes and value $\langle x \rangle =2$ represents case when all segments connect through both end nodes, effectively creating loops within the network. For example, the average number of nodes in segments in Fig. 2 is $\langle n \rangle =4$ and the crossover rate is $\langle x \rangle = 1.25$ since three segments connect with one end and one segment connects with both ends. The two merging nodes of different segments will form single node during the process of connection. Therefore, the total number of nodes present in the network dependent on event time will approximately be

2 $$N\left( t \right) \approx t\left( {\left\langle n \right\rangle - \left\langle x \right\rangle } \right) + \left\langle x \right\rangle .$$

The degree dynamics $k_i (t)$ depending on event time *t* was derived using analytically convenient continuum approximation and preferential attachment where we treat time *t* and degree *k* as continuous variables with increments *dt* and $dk_i$ respectively.

The probability $\Pi (k_i)$ of a newly added segment connecting to the node *i* with degree $k_i$ is

3 $$\Pi \left( {k_{i} } \right) = \frac{{k_{i} }}{{2t\left( {\left\langle n \right\rangle - 1} \right)}}$$

where $2t(\langle n \rangle -1)=\sum k_i$ . Using (3) it is possible to calculate the degree dynamics of node *i* added to the network at event time $t_i$ as

4 $$\begin{aligned} k_i=m\left( \frac{t}{t_i}\right) ^\beta \end{aligned}$$

where $m=k_i(t_i)$ is the initial degree of the node *i* and

5 $$\beta = \frac{{\left\langle x \right\rangle }}{{2\left\langle n \right\rangle - 2}}.$$

Using (4) one can derive degree distribution $p_k$ in continuum approximation to estimate the degree exponent $\gamma$ for PPA network properties estimation.

The resulting degree distribution $p_k$ is a power law distribution where $p_{k} \propto k^{{ - \left( {\frac{1}{\beta } + 1} \right)}}$. Comparing exponent $\gamma$ from power law degree distribution $p_k \propto k^{-\gamma }$ of scale-free networks^16,25^ and calculated degree distribution we obtain expected result where $\gamma =\frac{1}{\beta }+1$.

**Fig. 3:**
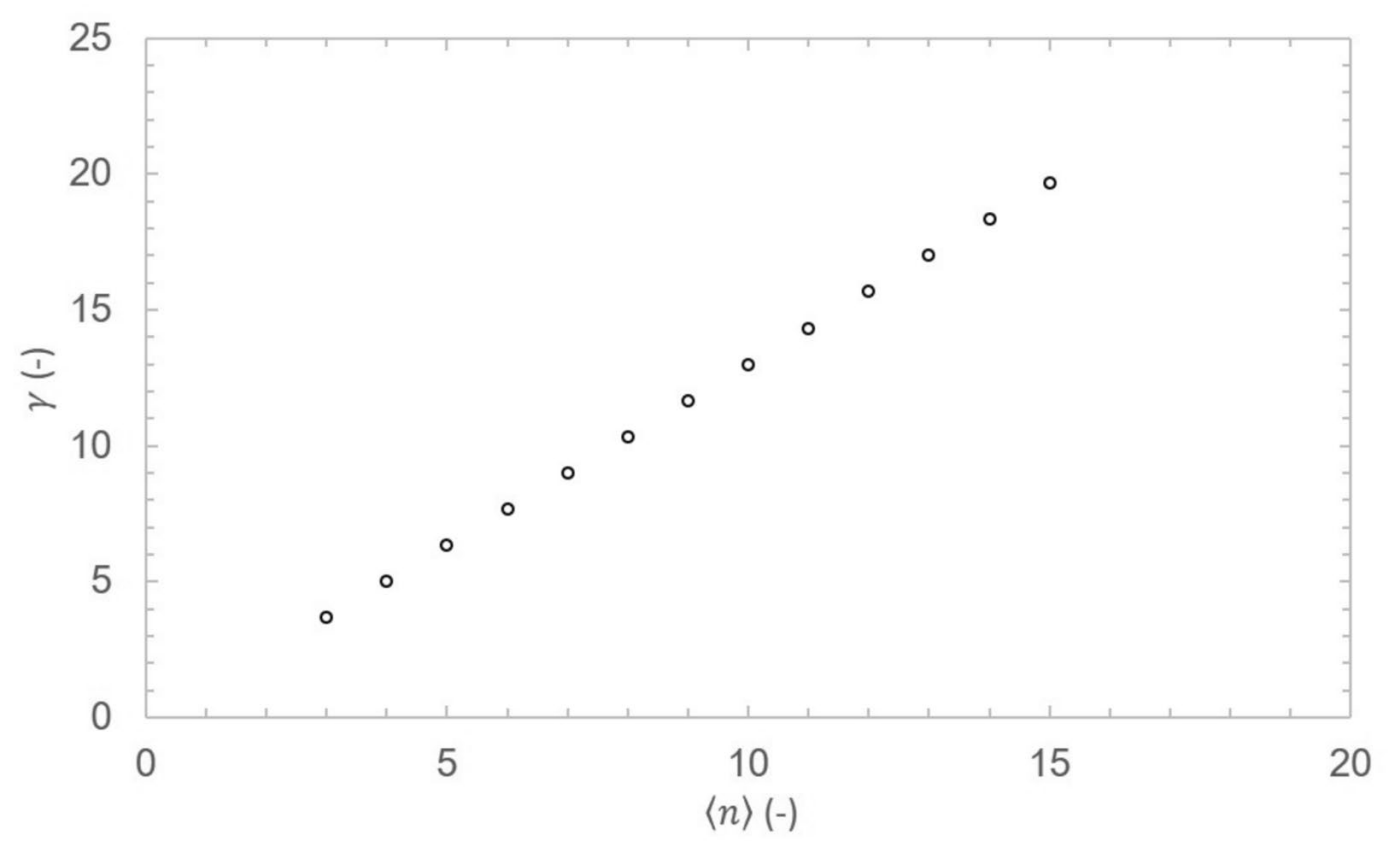
Degree exponent $\gamma$ as a function of average length $\langle n \rangle$ of path network segment with set average crossover rate to $\langle x \rangle =1.5.$.

In the case of path-based network, the degree exponent is $\gamma > 3$ for $\langle n \rangle > 3$ and constrained $\langle x \rangle$ as example is shown in Fig. 3. For the degree exponent $\gamma > 3$ the network is usually considered to operate with random network like properties (first and second moment of degree distribution is finite)^26,27^. In the case of $\langle n \rangle =3$ and $\langle x \rangle =2$ , the degree exponent $\gamma =3$ since $\beta = 2^{-1}$. The situation is equivalent to addition of one node with $m=2$ edges to the network. The resulting distribution then corresponds to Barabási-Albert (BA) model^16^. The continuum approximation does not provide exact degree distribution $p_k$ although it provides correct values of the degree exponent. Therefore, the exact $p_k$ is derived using rate equation method^28,29^.

To derive the degree distribution $p_k$ using rate equation method, we start from expected number of edges connecting to nodes with degree *k* after a new path segment arrives at event time *t* with average cross-over rate $\langle x \rangle$ as

6 $${\mathrm{number}}\;{\mathrm{of}}\;{\mathrm{edges}} = \Pi \left( k \right)N_{k} \left( t \right)\left\langle x \right\rangle$$

where $\Pi (k)$ is the probability of link formation and $N_k(t)=N(t) p_k (t)$ is the number of nodes with degree *k* where $p_k (t)$ is probability that randomly chosen node from the network will have degree *k* at event time *t*.

Under assumption that each of the nodes acquiring new edge changes its degree only by $\Delta k$ at any event time *t* increment, the number of nodes $N_k (t)$ increases with rate

7 $$N_{k}^{ + } \left( t \right) = \frac{{\left( {k - \Delta k} \right)\left\langle x \right\rangle \left[ {t\left( {\left\langle n \right\rangle - \left\langle x \right\rangle } \right) + \left\langle x \right\rangle } \right]}}{{2t\left( {\left\langle n \right\rangle - 1} \right)}}p_{{k - \Delta k}} \left( t \right).$$

Concurrently, the number of nodes with degree k decreases due to nodes gaining a link with rate

8 $$N_{k}^{ - } \left( t \right) = - \frac{{k\left\langle x \right\rangle \left[ {t\left( {\left\langle n \right\rangle - \left\langle x \right\rangle } \right) + \left\langle x \right\rangle } \right]}}{{2t\left( {\left\langle n \right\rangle - 1} \right)}}p_{k} \left( t \right).{\text{ }}$$

In total, the number of nodes with degree *k* at event time $t+1$ is

9 $$\left[ {N\left( t \right) + \left( {\left\langle n \right\rangle - \left\langle x \right\rangle } \right)} \right]p_{k} \left( {t + 1} \right) = N_{k} \left( t \right) + N_{k}^{ + } \left( t \right) + N_{k}^{ - } \left( t \right).$$

Number of nodes with elementary degree $k=m$ at event time $t+1$ is

10 $$\left[ {N\left( t \right) + \left( {\left\langle n \right\rangle - \left\langle x \right\rangle } \right)} \right]p_{m} \left( {t + 1} \right) = N_{m} \left( t \right) + N_{m}^{ + } + N_{m}^{ - } \left( t \right).$$

where $p_m (t)$ is the probability that randomly chosen node from the network will have degree *m* and $N_m (t)$ is number of nodes with degree *m*.

The term

11 $$N_{m}^{ + } = \left\langle n \right\rangle - \left\langle x \right\rangle .$$

represents the source of nodes arriving at degree *m*. Since each arriving path segment consists primarily of interior nodes $(n_i-2)$ with degree $k = 2$, we approximate all new nodes as arriving at the degree $k = m = 2$. The end nodes with degree $k = 1$ require separate treatment due to their distinct dynamics (see (17)-(20)).

The reduction rate of number of nodes $N_m (t)$ with degree *m* is represented as

12 $$N_{m}^{ - } \left( t \right) = - \frac{{m\left\langle x \right\rangle \left[ {t\left( {\left\langle n \right\rangle - \left\langle x \right\rangle } \right) + \left\langle x \right\rangle } \right]}}{{2t\left( {\left\langle n \right\rangle - 1} \right)}}p_{m} \left( t \right).$$

Although the choice of minimum degree *m* affects the distribution, the final normalized probabilities $p_{k}^{\text {norm}}$ are independent of *m* (see (21)).

Moving the $N_k (t)$ and $N_m (t)$ to the l.h.s. of (9) and (10), respectively, and operating in asymptotic regime, where $t \rightarrow \infty$, the probability $p_k (t) \rightarrow p_k$ and $p_k (t+1) \rightarrow p_k$ and similarly for $p_m (t) \rightarrow p_m$ and $p_m (t+1) \rightarrow p_m$ the (9) and (10) becomes

13 $$\left( {\left\langle n \right\rangle - \left\langle x \right\rangle } \right)p_{k} = N_{k}^{ + } \left( \infty \right) + N_{k}^{ - } \left( \infty \right)$$

and

14 $$\left( {\left\langle n \right\rangle - \left\langle x \right\rangle } \right)p_{m} = N_{m}^{ + } + N_{m}^{ - } \left( \infty \right),$$

respectively, since $p_k (t+1)-p_k (t) \rightarrow p_k-p_k=0$ which represents a steady state of the network. Similarly, for $p_m$.

Rewriting $k \rightarrow k+\Delta k$ the resulting rate equations are

15 $$p_{{k + \Delta k}} = \frac{{k\left\langle x \right\rangle }}{{2\left( {\left\langle n \right\rangle - 1} \right) + \left( {k + \Delta k} \right)\left\langle x \right\rangle }}p_{k}$$

and

16 $$p_{m} = \frac{{2\left( {\left\langle n \right\rangle - 1} \right)}}{{2\left( {\left\langle n \right\rangle - 1} \right) + m\left\langle x \right\rangle }}.$$

We obtain probability $p_m$ directly from (16). Using (15) and (16) we can iteratively calculate the degree distribution $p_k$ for $k \in \{m+\Delta k, m+2\Delta k, \ldots \}$, where $\Delta k=1$, of PPA network depending on average segment length $\langle n \rangle$ and crossover rate $\langle x \rangle$.

The resulting degree distribution $p_k$ applies, however, to $k \ge 2$ and probability $p_1$ cannot be calculated using this method. End nodes with degree $k = 1$ follow different dynamics from interior nodes. Since rate equations cannot be directly applied, we derived probability $p_1 (t)$ considering two limiting cases and taking their weighted average. The first scenario assumes the case when all newly added segments connect to nodes with degree $k \ge 2$ and number $N_1$ of nodes with degree $k=1$ grows with event time as $N_{1}^{w_1}(t)= (t - 1)(2 - \langle x \rangle )+2$. In another extreme case, the newly added segments connect to nodes with degree $k = 1$ and number of nodes with degree $k = 1$ is constant in time as $N_{1}^{w_2}=2$. The resulting network is then equivalent to the single path.

**Fig. 4:**
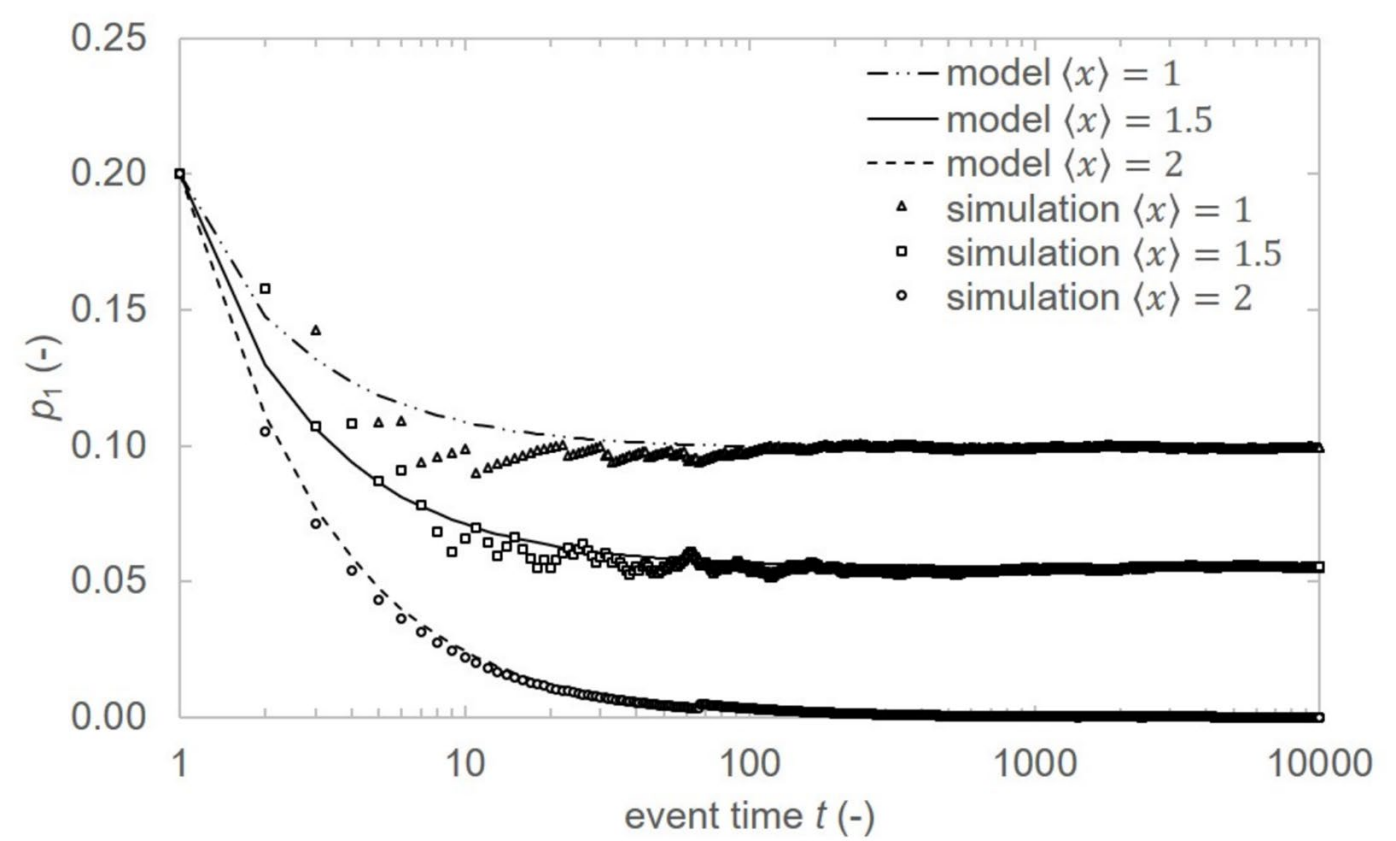
Evolution of $p_1 (t)$ with event time *t* for $\langle n \rangle =10$, varying crossover rate $\langle x \rangle$. Lines: analytical model; symbols: simulations.

The resulting probability that randomly chosen node will have degree $k=1$ is

17 $$p_{1} \left( t \right) = w_{1} \left( t \right)\frac{{N_{1}^{{w_{1} }} \left( t \right)}}{{N\left( t \right)}} + w_{2} \left( t \right)\frac{{N_{1}^{{w_{2} }} }}{{N\left( t \right)}}$$

where

18 $$w_{1} \left( t \right) = \frac{{\left( {t - 1} \right)\left( {\left\langle n \right\rangle - 2} \right)}}{{\left( {t - 1} \right)\left( {\left\langle n \right\rangle - \left\langle x \right\rangle } \right) + \left\langle x \right\rangle }}$$

and

19 $$w_{2} \left( t \right) = 1 - w_{1} \left( t \right)$$

are the respective weights of the probabilities $p_{1}^{w_1}(t) = \frac{N_{1}^{w_1}(t)}{N(t)}$ and $p_{1}^{w_2}(t) = \frac{N_{1}^{w_2}}{N(t)}$. The evolution of probability $p_1 (t)$ in event time was simulated and comparison of the model and single run of discrete simulation is in Fig. 4. All cases exhibit convergence from initial values to steady states for event time $t>100$ and the simulation result corresponds well to the predicted value. Probability $p_1 (t)$ decreases with increasing $\langle x \rangle$ as expected since higher crossover rate results in lower number of isolated end nodes.

For $t \rightarrow \infty$ the probability $p_1 (t) \rightarrow p_1$ of randomly chosen node having degree $k=1$ becomes

20 $$p_{1} = \frac{{\left( {\left\langle n \right\rangle - 2} \right)\left( {2 - \left\langle x \right\rangle } \right)}}{{\left( {\left\langle n \right\rangle - \left\langle x \right\rangle } \right)^{2} }}.$$

The addition of probability $p_1$ to the degree distribution $p_k$ results in the need to normalize the probabilities for $k \ge 2$ to satisfy $\sum p_k = 1$. Probability $p_1$ is already normalized and only $p_k$ for $k \ge 2$ remains to be adjusted. The normalized probability for $k \ge 2$ is then

21 $$\begin{aligned} p_{k \ge 2}^{\text {norm}} = \frac{1-p_1}{\sum _{k=2} p_k}p_{k \ge 2}. \end{aligned}$$

The normalization procedure in (21) naturally accounts for the minimum degree parameter *m*. Since the degree distribution is calculated as $p_k=f(p_m)$ dividing by $\sum _{k=2}p_k$ eliminates the dependence on *m*.

### Barabási-Albert model equivalence validation

The BA model can be directly obtained using $m= \langle x \rangle$ where $\langle x \rangle \in \mathbb {N}$ and imposing condition for $\langle x \rangle$ as

22 $$\begin{aligned} \langle x \rangle = \langle n \rangle - 1. \end{aligned}$$

Using (5) one can rewrite (16) to

23 $$\begin{aligned} p_m = \frac{1}{1+m\beta }. \end{aligned}$$

**Fig. 5:**
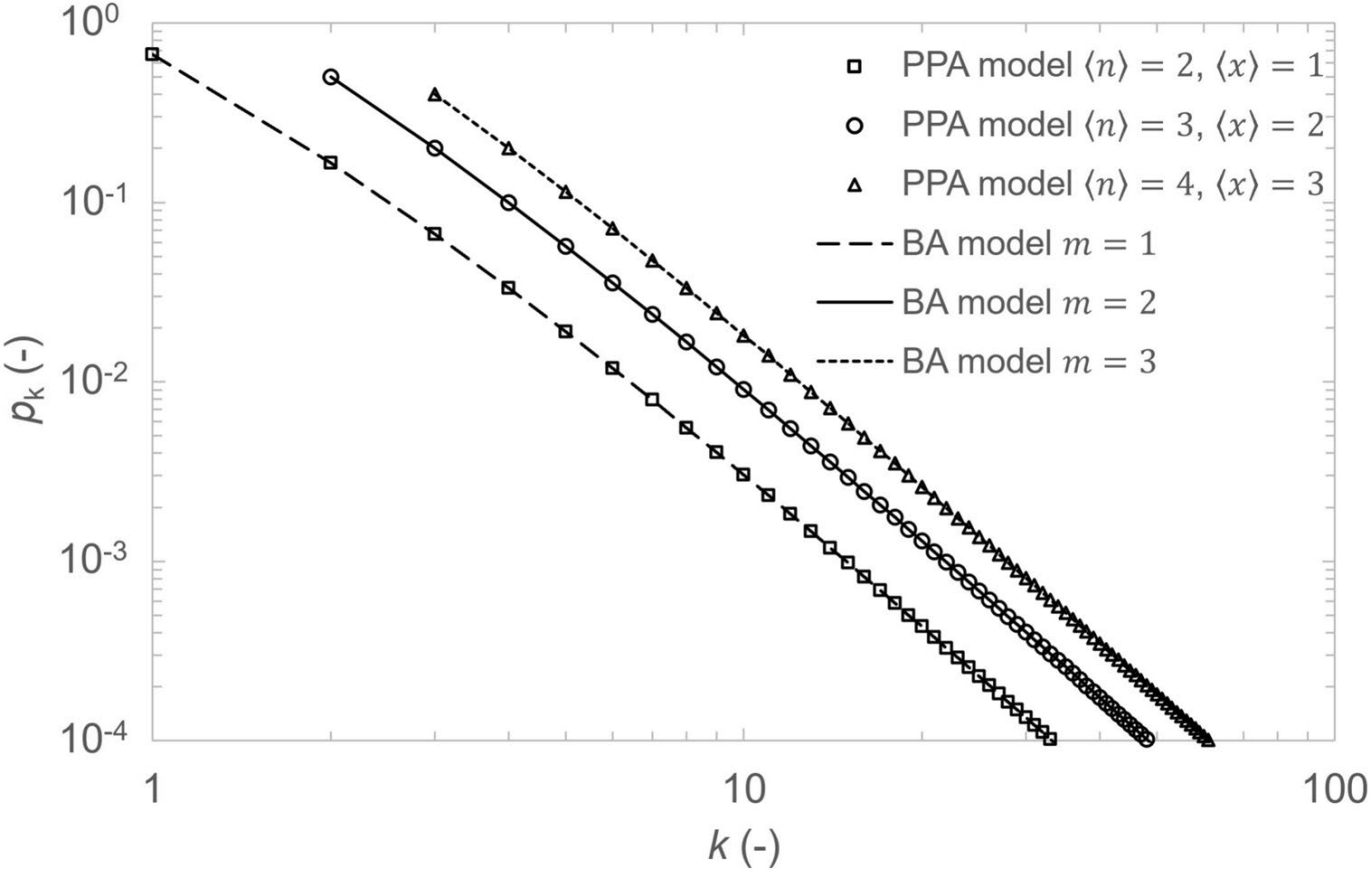
Degree distributions comparing PPA model (symbols) with standard BA model (lines) for $m = 1,2,3$. Using the $\langle x \rangle = \langle n \rangle - 1$ condition, distributions overlap exactly, validating analytical equivalence. All show $\gamma = 3$ power-law scaling. Realistic QKD parameters $\langle x \rangle < \langle n \rangle - 1$ deviate from this condition, producing $\gamma > 3$.

If $\beta =\frac{1}{2}$ (true for $\langle x \rangle = \langle n \rangle - 1$ condition) we directly obtain $p_m$ in BA model. Similarly, the (15) can be rewritten to the form of BA result using the same bounding condition (Fig. 5).

### Network simulation

To further examine some properties of our PPA model, the network model was implemented in Python using *NetworkX* library^30^.

The network core implementation incorporates random path length $n_i$ generation with a normal distribution for which the mean value and standard deviation are $\langle n \rangle$ and $\sigma _\text {n}=\frac{\langle n \rangle -n_{\text {min}}}{3}$, respectively, where $n_{\text {min}}=2$ is the minimum number of nodes and $n_{\text {max}}= 2 \langle n \rangle -n_{\text {min}}$ is the maximum number of nodes in a new arriving path. This follows the three-sigma rule, ensuring approximately $99.7 \%$ of generated path lengths $n_i$ fall within the range $[n_{\text {min}}, n_{\text {max}}]$, symmetrical around the mean value. Random uniform selection of parameter $x_i$ deciding whether new path merges one end node or both end nodes with existing nodes is implemented. Preferential attachment approach for new path connection and manually adjustable number of instances when only an edge is added to the network creating shortcut connection between two random, already existing nodes is incorporated. The edge addition effectively simulates satellite link being created between selected nodes. The networks robustness and average distance is compared for various number of edges (satellite links).

**Fig. 6:**
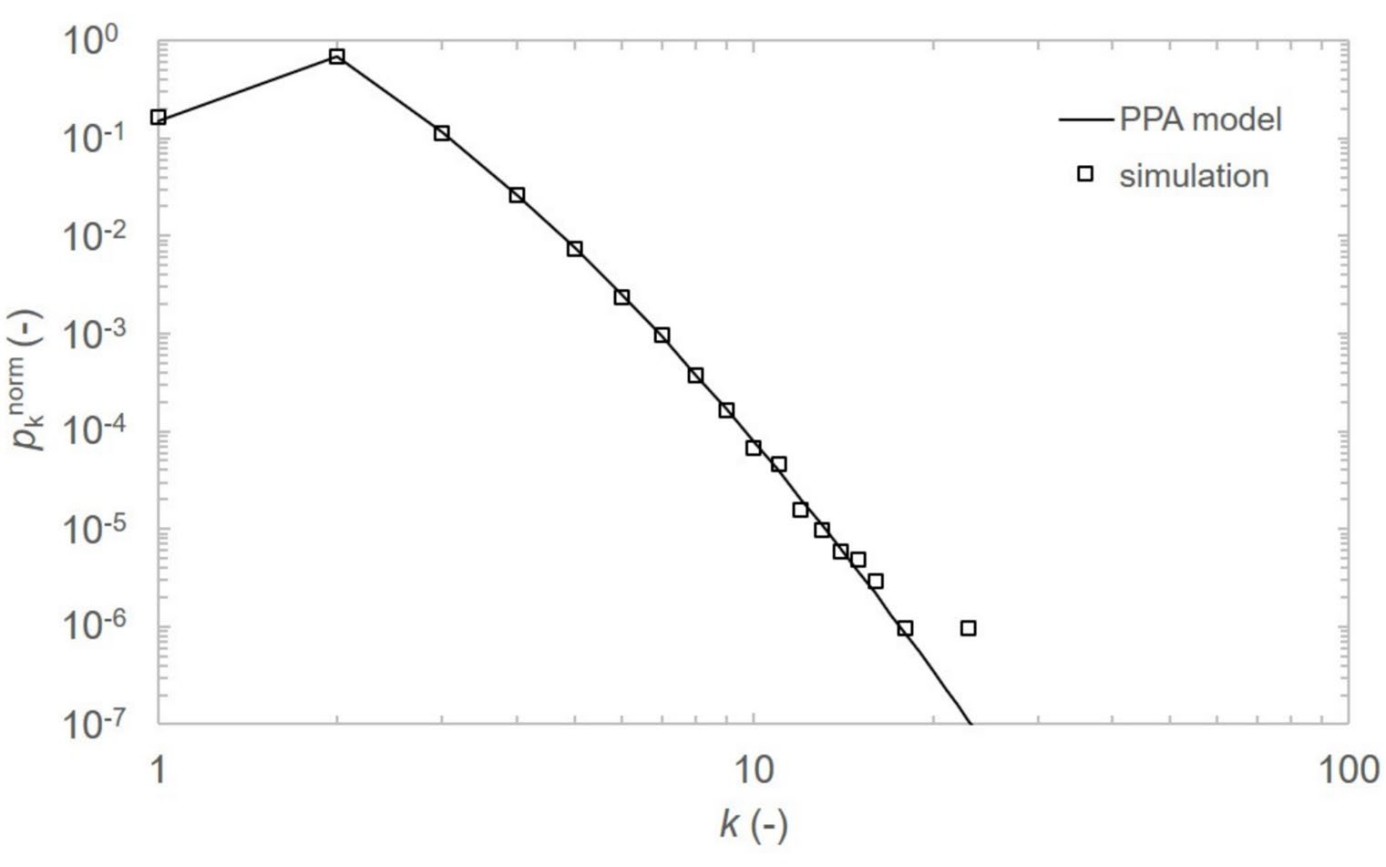
Degree distribution $p_{{k}}^{\text {norm}}$ for average crossover rate $\langle x \rangle = 1.10$ and number of nodes within path segment $\langle n \rangle = 6$. Comparison between model (solid line, $N \rightarrow \infty$) and computer simulation (squares) for $N = 1032109$ nodes. The slight disagreement in $p_{k}^{\text {norm}}$ for $k \ge 10$ is due to finite-size simulation network effect.

To assess the correctness of our model implementation, both the analytical degree distribution $p_{k}^{\text {norm}}$ and degree distribution from simulated network are compared and presented in Fig. 6. Simulated data is represented by squares and mathematical model is represented by solid line for $\langle n \rangle = 6$, $\langle x \rangle = 1.1$ and $N = 1032109$ nodes. The distribution experiences low degree saturation and high degree cutoff, a phenomenon observed in real-life networks^16,31^.

**Fig. 7:**
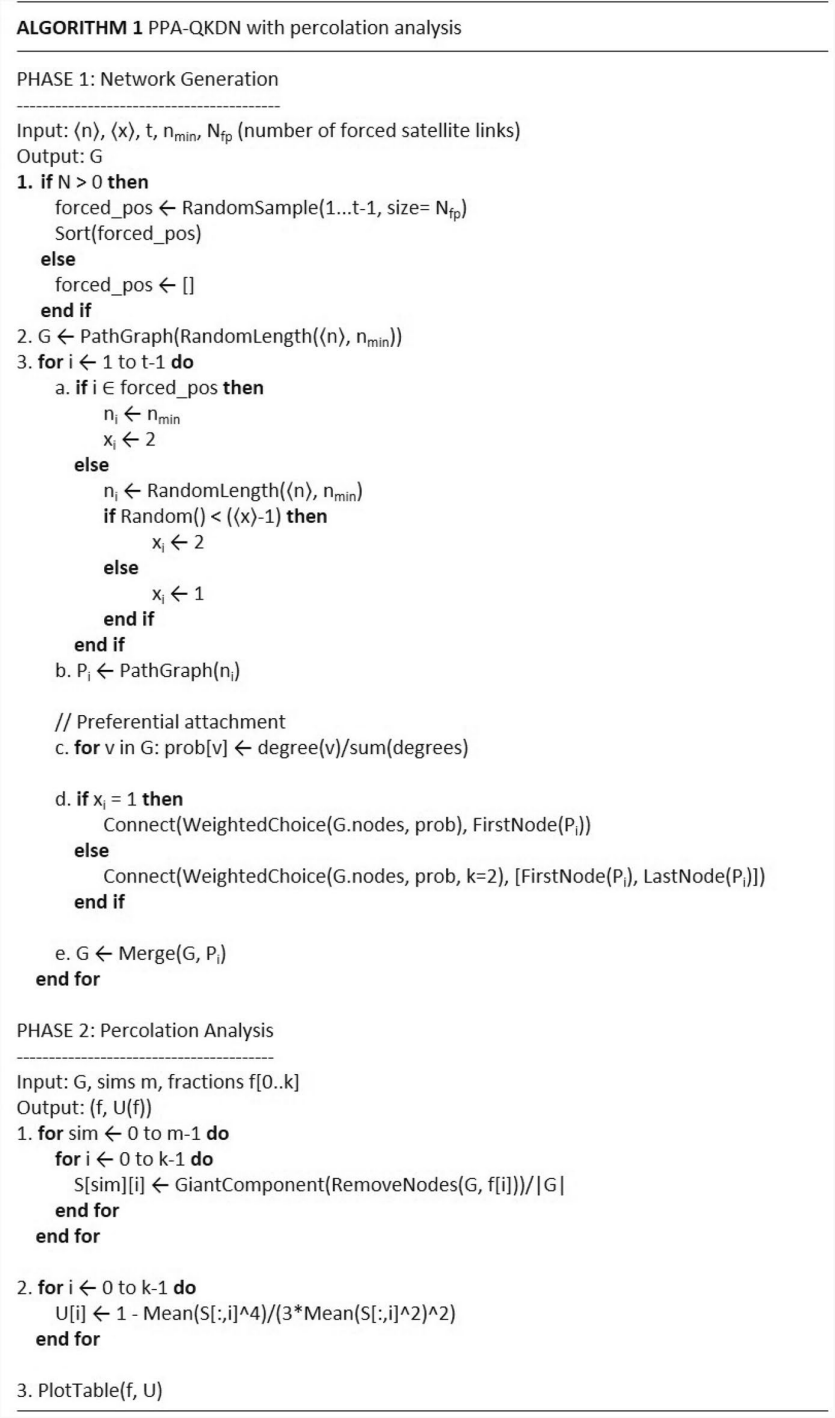
Pseudocode of the PPA network formation process and percolation analysis.

The pseudocode is shown in Fig. 7. It shows basic blocks of Python implementation of the PPA model. The first block – *Phase 1* describes network formation. It incorporates random path length generation (normally distributed) and path graph merging driven by preferential attachment. Moreover, the satellite link formation is represented by number $N_{fp}$ of forced positions *forced_pos* where only an edge is placed in the network connecting two randomly selected nodes.

Second block – *Phase 2* analyzes the network robustness by calculating (normalized) giant component (GC) size for each fraction f of nodes removed from network. This serves as input for Binder cumulant^32^ calculation. Network robustness i.e. the ability to remain functional despite removing nodes from the network constitutes important metric to consider while designing networks topology.

## Results

### Network robustness analysis

The network robustness was studied through iterative node removal process for multiple network realizations. For each selected parameter $\langle n \rangle$ and $\langle x \rangle$, 50 network realizations were simulated. For each particular network realization 50 node removal processes were performed resulting in 2500 cases overall. A size of GC was measured and normalized for each fraction *f* of removed nodes during the node removal process. The GC represents the largest cluster of connected nodes within the network that scales with number of nodes *N*. All nodes in the network are part of the GC in context of preferentially attached QKDN, since $x_i\ge 1$ for every $i-th$ path meaning every new path created connects to node(s) in the network. The network is connected and therefore, any pair of nodes in QKDN can establish a path for secure communication. As the nodes are randomly removed the network fragments and loses the GC eventually. The critical fraction $f_\text {c}$ of nodes removed from network represents a threshold for the GC loss where network experiences phase transition. Network maintains its GC for $f<f_\text {c}$ and in the case when $f>f_\text {c}$ the network fragments into isolated clusters of nodes. Higher $f_\text {c}$ values result in robust networks, e.g. scale-free networks ($2<\gamma <3$) tend to have $f_\text {c} \rightarrow 1$ for large size networks^33^.

**Fig. 8:**
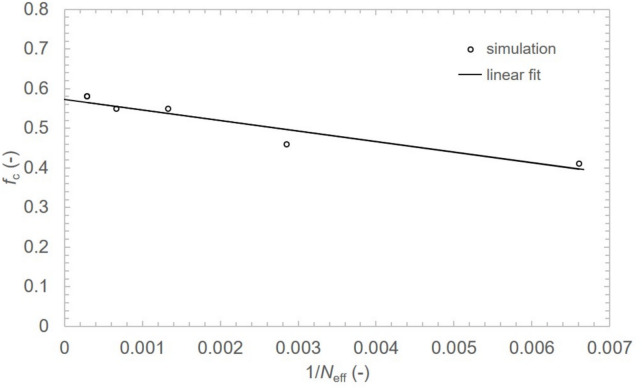
Network robustness (critical fraction $f_\text {c}$) dependent on effective number of nodes $N_\text {eff}$ for $\langle n \rangle = 4$, $\langle x \rangle = 1.1$ and number of satellite links $n_\text {sat} = 0.2t$.

To identify the phase transition in the finite size QKDNs and accurately determine the $f_\text {c}$ value, the Binder cumulant U was calculated for a set of $\langle n \rangle = \{4,6\}$, $\langle x \rangle = \{1.00,1.10,1.25\}$ and $r=\{0,0.1,0.2\}$. The ratio r of satellite links versus number of path graphs connected to the network allows us to calculate a number of satellite links as $n_\text {sat}= rt$. For example, $r=0.1$ represents creating one satellite link per 10 terrestrial paths. The individual critical fractions $f_\text {c}$ were acquired by pairwise intersection analysis of Binder cumulant curves for two consecutive network sizes $N_i$ and $N_{i+1}$ where $N \in \{100, 200, 500, 1000, 2000, 5000\}$. The $f_\text {c}$ is plotted against $\frac{1}{N_\text {eff}}= \frac{2}{N_i+N_{i+1}}$. The critical fraction corresponding to $f_\text {c}$ ($N_\text {eff} \rightarrow \infty$) is obtained by extrapolating the linear fit ($R^2=0.75-0.97$) of the individual critical fractions of finite networks and finding an intersection with *y* axis of the plot^34^. Generally, $f_\text {c}$ increases as $\frac{1}{N_\text {eff}}$ decreases due to transition of network topology from tree-like to random graph behavior for larger networks as in example shown in Fig. 8.

**Table 1:** Critical fraction $f_{\text {c}}$ of PPA network for $\langle n \rangle =4$ and 6.

$\langle n \rangle = 4/6$	$n_\text {sat}=0$	$n_\text {sat}=0.1t$	$n_\text {sat}=0.2t$
$\langle x \rangle = 1.00$	$-/-$	0.46/0.33	0.54/0.40
$\langle x \rangle = 1.10$	0.46/0.26	0.53/0.36	0.57/0.43
$\langle x \rangle = 1.25$	0.53/0.34	0.59/0.42	0.64/0.48

The $-/-$ entries for $\langle x \rangle = 1.00$ and $n_{\text {sat}}=0$ indicate cases where Binder cumulant analysis could not determine $f_{\text {c}}$ due to nearly identical curves in the tree regime of the simulated network.

The resulting critical fraction for node removal in thermodynamic limit $f_\text {c}$ ($N_\text {eff} \rightarrow \infty$) is shown in Table 1. The Binder cumulant curves for different network sizes are nearly identical for $\langle x \rangle = 1.0$ (tree regime), preventing detection of intersections (data points in Fig. 8) to determine $f_\text {c}$ using Binder cumulant analysis. The network robustness increases with $\langle x \rangle$ as loops form within the network. The network robustness also increases with the number of satellite links $n_\text {sat}$ as expected, creating bridges between random parts of network. The $f_\text {c}$ value for $\langle n \rangle =6$ are generally smaller that for networks with $\langle n \rangle =4$. This is due to increased average length of individual network segments and therefore, more path-like behavior.

### Average distance and random network regime

Average distance describes how far are individual users of the network from each other on average. The ability to calculate and optimize average distance in future QKD networks is crucial for correct and proper financial settings of key relaying economy and, simultaneously, can reduce the key consumption in the relaying process.

**Fig. 9:**
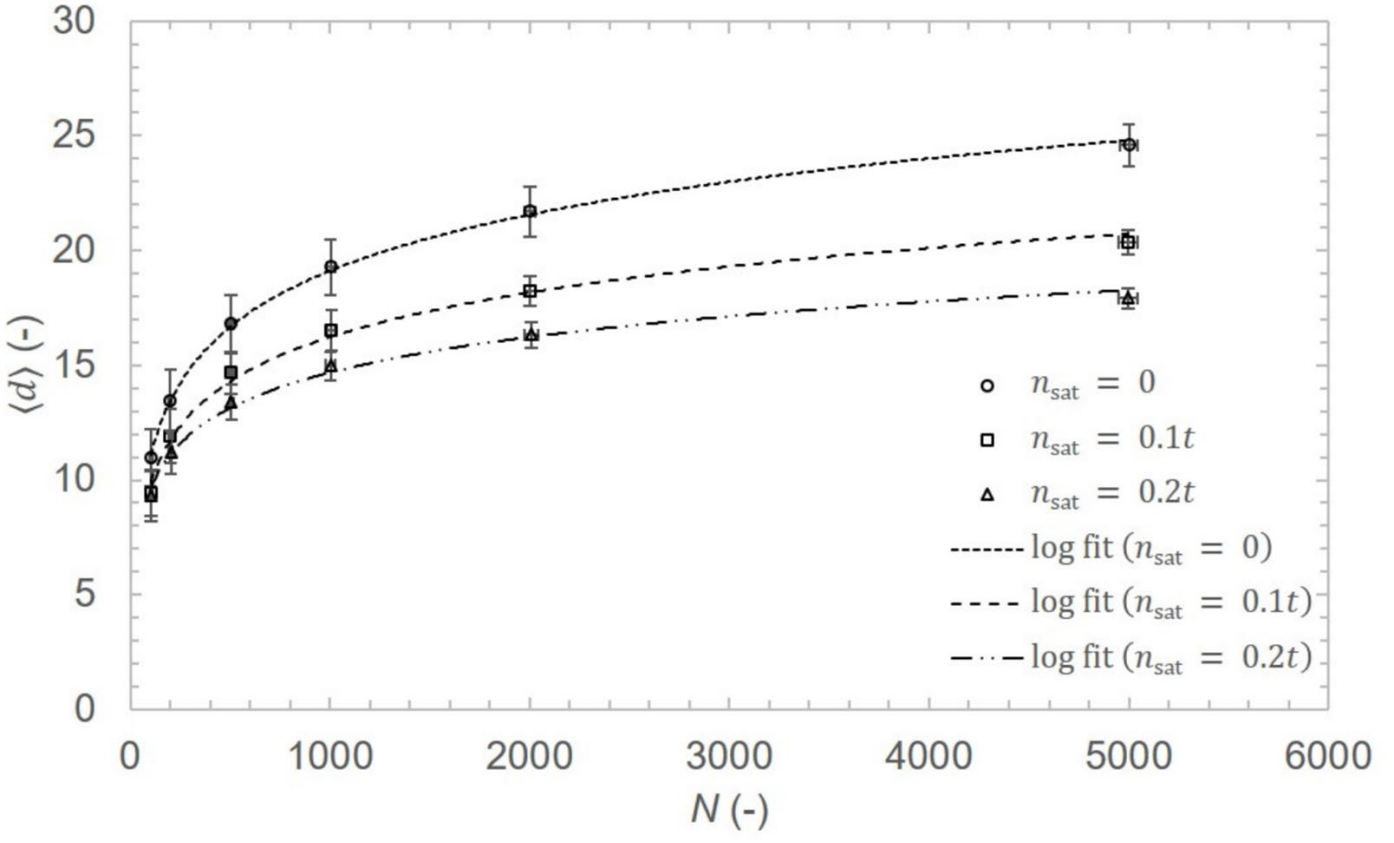
Average distance $\langle d \rangle$ in the preferential path attachment model with $\langle n \rangle = 6$ and $\langle x \rangle = 1.1$ for three shortcut densities: $n_\text {sat}=0$ (dotted line), $n_\text {sat}=0.1t$ (dashed line), and $n_\text {sat}=0.2t$ (dash-dot line). Markers represent simulation data with error bars. The average distance scales with the number of nodes as $\langle d \rangle \propto \ln (N)$ for all cases, with $R^2 > 0.99$.

Average distances between network nodes for simulated network realizations were obtained with *average_shortest_path_length* algorithm from the *NetworkX* library. The resulting dependence of average distance $\langle d \rangle$ on network size *N* is $\langle d \rangle \propto \ln (N)$ ($R^2 > 0.99$) for all combinations of $\langle n \rangle \in \{4, 6\}$, $\langle x \rangle \in \{1.0, 1.1, 1.25\}$, and $n_\text {sat} \in \{0, 0.1t, 0.2t\}$, as shown in Fig. 9. This further indicates the PPA model falls into the class of random networks, as the average distance in Erdös-Rényi random networks scales with the number of nodes *N* as $\langle d \rangle \propto \ln (N)$^35^, rather than the $\ln (\ln (N))$ scaling of scale-free networks. This quantifies the efficiency penalty of path-constrained infrastructure deployment.

### Satellite links finite economic advantages

**Fig. 10:**
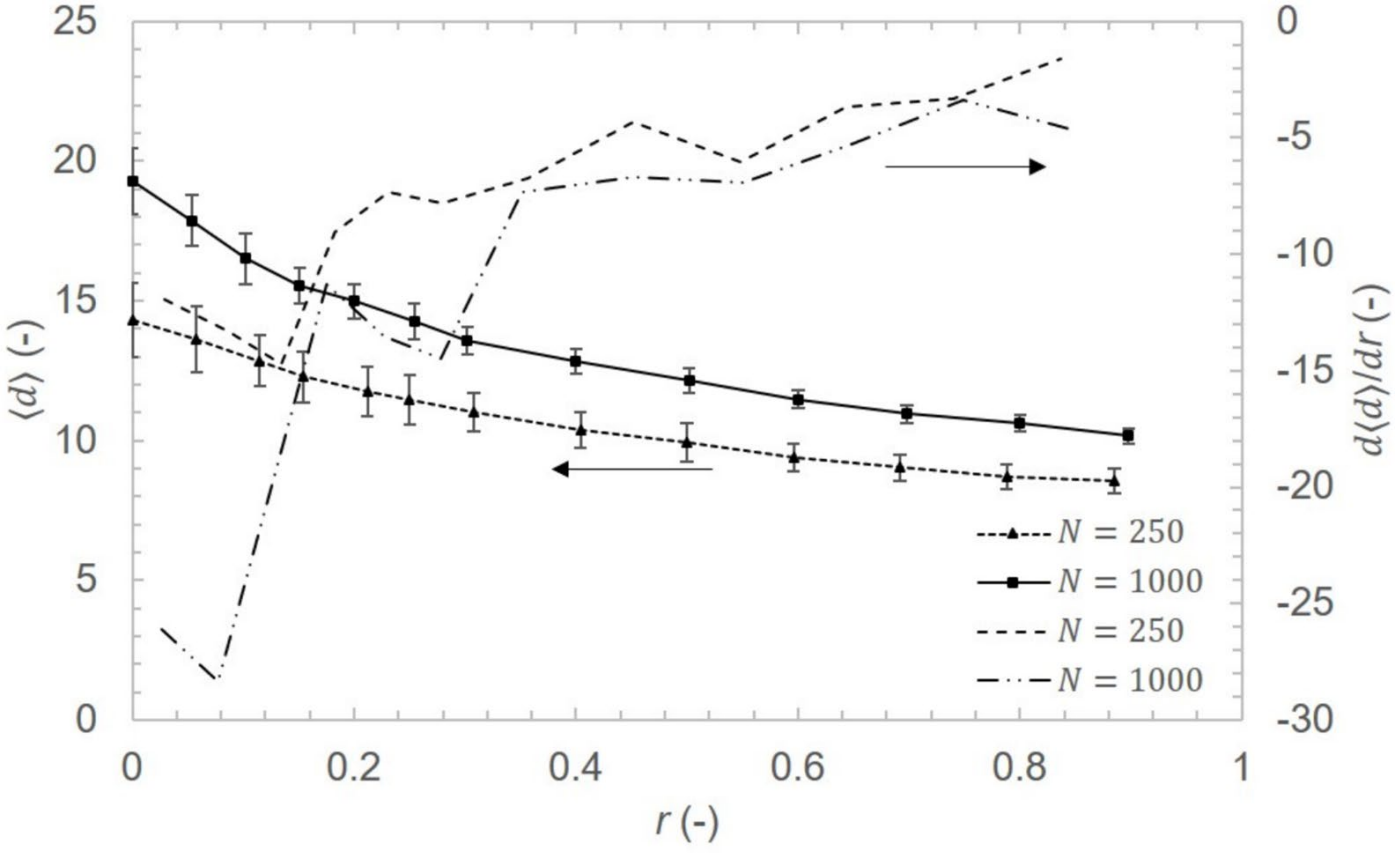
Satellite link effect on average distance between network nodes for $\langle n \rangle = 6$ and $\langle x \rangle = 1.1$. First derivative $\frac{d\langle d \rangle }{dr}$ shows larger impact on average distance for approx. $r \le 0.3$.

An analysis of satellite link effect on network’s average distance $\langle d \rangle$ was performed. Results for $\langle n \rangle =6$ and $\langle x \rangle = 1.1$ are shown in Fig. 10. Increasing the fraction r of satellite links leads to initial decrease in average distance $\langle d \rangle$. Nevertheless, the slope of average distance decrement tends to be smaller as the fraction of satellite links r increases. For $r > 0.3$ the effect on average distance is less pronounced and economic benefits should be thoroughly assessed.

The functional role of satellite links in reducing average distance is analogous to the Watts-Strogatz rewiring mechanism^35^. However, contrary to link rewiring, our implementation adds new connections without removing existing infrastructure. This, in our opinion, should better reflect reality where satellite links, express routes, or transmission lines supplement rather than replace underlying network structure.

### Key consumption scaling with network topology

We estimate the dependence of the overall expected secret key bit consumption rate *SKCR* on the average distance $\langle d \rangle$ due to trusted node relaying in undirected (communication in both directions, unweighted) QKDN as

24 $$\begin{aligned} SKCR = N(N-1)L_{\text {k}}R_{\text {k}}(\langle d \rangle - 1)f_{\text {active}} \end{aligned}$$

where *N* is the number of nodes in network, $L_{\text {k}}$ is the key length (256 bits for AES-256) used for plaintext encryption, $R_{\text {k}}$ is the key refresh rate per second ($R_{\text {k}}=const.$ for all $d_{ij}$ for simplicity), $\langle d \rangle = \frac{\sum d_{ij}}{N(N-1)}$ for every pair *i*, *j* where $i\ne j$ (undirected and unweighted edges^35^). The parameter $f_{\text {active}}$ represents the fraction of all node pairs *i*, *j* engaged in simultaneous communication through the shortest path and is treated as a topology-independent constant, so that the scaling comparison across network classes in (24) is governed only by $\langle d \rangle$. For overall *SKCR* in QKDN (key relaying and key for data encryption/decryption) we would omit the ‘$-1$’ in $(\langle d \rangle -1)$.

**Fig. 11:**
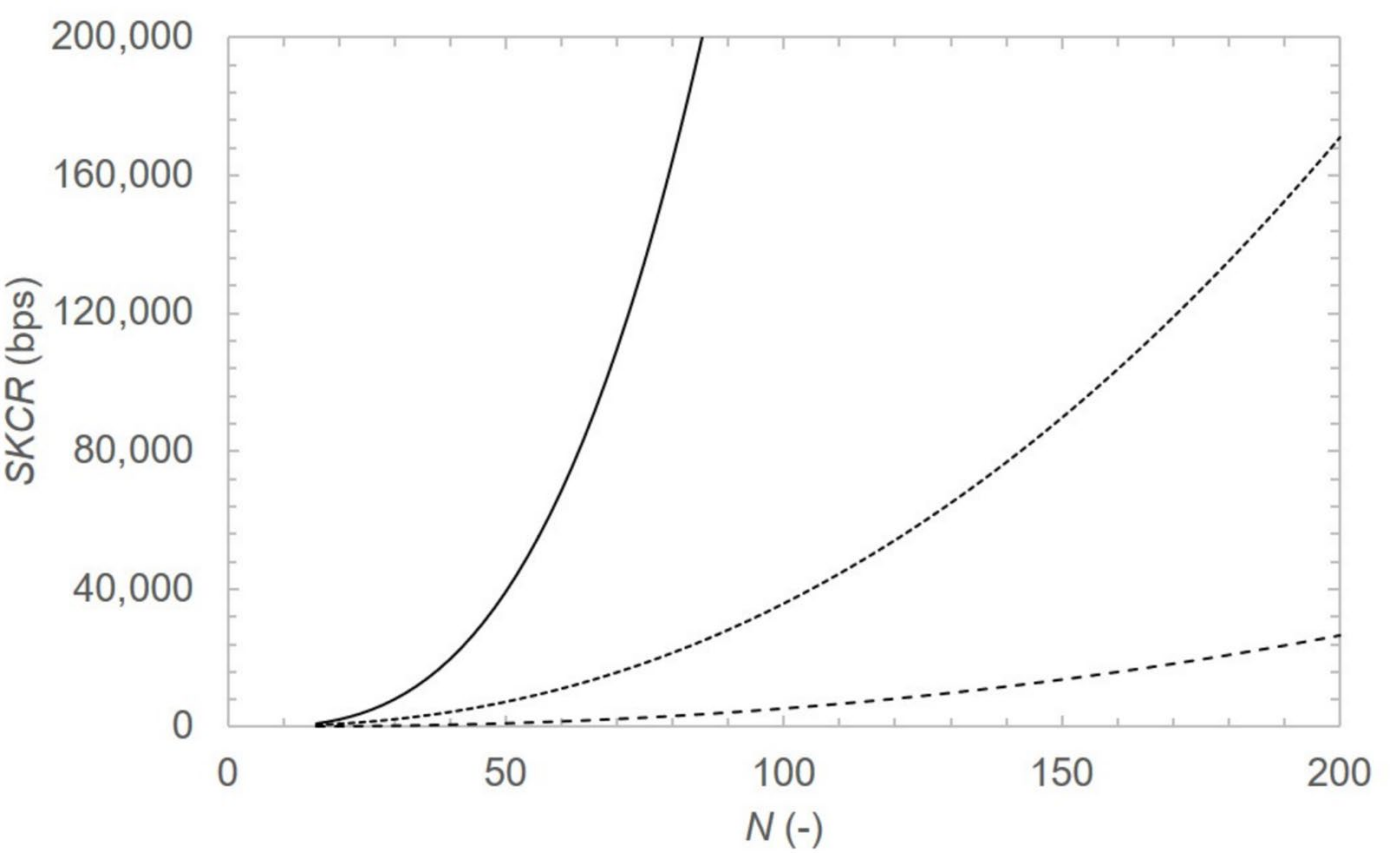
Secret key bit consumption rate due to key relaying processes per unit encryption key length $L_{\text {k}}$ and unit key refresh rate $R_{\text {k}}$ dependent on number of nodes *N* in simple path network (solid line), random network (dotted line), where $\langle d \rangle \propto \ln (N)$, and scale-free network (dashed line), where $\langle d \rangle \propto \ln (\ln (N))$. Fraction $f_{\text {active}}=1$ used in the analysis means all node pairs contribute to the *SKCR*.

Figure 11 shows approximated *SKCR* dependence on number of nodes *N* in different classes of network. Scale-free networks^25^ exhibit lowest *SKCR* due to the $\langle d \rangle \propto \ln (\ln (N))$^36^ dependence of average distance $\langle d \rangle$ on number of nodes *N*. Following the results in Fig. 11, it is desirable to reduce the scaling of average distance $\langle d \rangle$ with *N*. A topology of QKDN similar to scale-free networks is therefore favorable.

## Discussion

In this study, we proposed a PPA model of QKDN with preferential path attachment. The exact degree distribution of path-based network was derived using the rate equation method leading to formulation of a normalized degree distribution characterized by saturation in low-degree nodes and a prominent cutoff in high-degree nodes. The agreement between analytical predictions (lines) and simulation results (symbols) across the varying crossover rate $\langle x \rangle$ validates our dual analytical framework for $p_1$ derivation (Fig. 4) combined with rate equations for $k\ge 2$ for path attachment network formation process. We demonstrated model equivalency to the BA model, exactly reproducing the degree distributions when path constraints vanish.

To validate the theoretical predictions, a network model was implemented in Python, and its correctness was rigorously assessed against the analytical degree distribution. Utilizing a network simulator, we extensively analyzed network properties with a focus on robustness and path length between node pairs. The robustness of the path-based network was quantitatively evaluated using Binder cumulants and extrapolated to the large network limit. Our findings reveal that the critical fraction of node removal required to disrupt the giant cluster decreases as the average segment length $\langle n \rangle$ increases. This observed trend aligns with expectations as the network adopts enhanced path-like structural characteristics. Satellite links tend to increase the critical fraction $f_{\text {c}}$ beyond which the network fragments and loses its giant component. This behavior is expected as loops are formed within the QKDN.

In practice, ground station placement for satellite link establishment is subject to geographical constraints while the signal transmission is subject to temporal constraints affected by atmospheric conditions and orbital geometry. The random shortcut placement used here is a topological approximation. Incorporating geographical constraints on both node and link placement is expected to affect the efficiency of shortcuts and average distance between nodes.

The PPA model analysis reveals that path constraints prevent hub formation despite preferential attachment, resulting in a degree distribution with $\gamma > 3$. The average distance between nodes in QKDN exhibits logarithmic dependence $O(\log (N))$ on network size *N* for the studied range of average path segment lengths $\langle n \rangle$, crossover parameters $\langle x \rangle$, and numbers of satellite links $n_{\text {sat}}$. Increasing the number of satellite links $n_{\text {sat}}$ decreases the average distance $\langle d \rangle$ by creating long-range interconnections. Nevertheless, the decreasing effect on the average distance diminishes as the fraction of satellite links increases. These findings reveal important limitations for QKD network planning. First, path constraints fundamentally limit topology optimization as hub formation is prevented despite preferential attachment. Second, satellite links exhibit diminishing returns. Network designers cannot rely on either mechanism to arbitrarily minimize the average distance for efficient key relaying. The average distance derived here provides the necessary infrastructure-level input for resource scheduling in QKDNs, as it directly determines the number of links occupied per key distribution session. Each session occupies on average $\langle d \rangle$ links, therefore, the logarithmic scaling $\langle d \rangle \propto \ln (N)$ limits the number of links shared between concurrent sessions compared to networks with *O*(*N*) average distance scaling, reducing contention for finite key resources. Furthermore, the degree exponent $\gamma > 3$ implies that large hubs practically do not form, distributing key demand more uniformly across the network in contrast to networks with $\gamma \le 3$ where hubs concentrate concurrent sessions on adjacent links.

Current long-haul QKDN deployments, including recent large-scale networks^1,22^, remain limited to single or few chain segments, representing early-stage cases where the topological properties predicted by the PPA model have not yet emerged or have only begun to emerge. Planned complex large-scale infrastructures such as EuroQCI^23^, designed to interconnect national quantum networks across Europe, are expected to reach scales where rigorous validation of the PPA model predictions becomes feasible. Furthermore, recent advances in long-distance QKD^37,38^ effectively reduce the required segment length $\langle n \rangle$, which per Fig. 3 decreases $\gamma$ toward 3 and may enable more favorable topological properties. In contrast, multi-party protocols including quantum conference key agreement^39,40^ and quantum secret sharing^41^ represent emerging applications that would require fundamentally different network formation models.

While our analysis focuses on quantum communication networks, the PPA model applies to any path-based infrastructure where new segments preferentially attach to existing nodes and merging creates composite structures.

Future work could explore how different QKD technologies e.g., entanglement-based technology^42^ and alternative network architectures affect scalability of QKDNs and average distance between nodes, creating a promising direction for advancing quantum secure communications beyond path-constrained designs.

## Data Availability

The simulation code and datasets generated during this study are available from the corresponding author upon reasonable request.
